# On the Use of the Pad in Varicose Ulcers

**Published:** 1855-08

**Authors:** R. Barnwell

**Affiliations:** Assistant Surgeon at Charing Cross Hospital


*On the Use of the Pad in Varicose Ulcers.* By R. BARNWELL, F.R.C.S. Assistant Surgeon at Charing Cross Hospital.

The result of some cases of varicose ulcer, treated on a plan which, though not new, is scarcely sufficiently well known, may be of interest. One case, the worst I have thus treated, will be sufficient. It occurred in the person of a poor woman, aged 58; and the ulcer on the ancle was deep, surrounded by blue hard edges, was extremely painful, and discharged, in considerable quantity, a thin pus. It had been kept bandaged, treated with various lotions and ointments, for two years, and the foot had remained so perfectly rigid, for fear of the pain on moving, that a partial anchylosis appeared to have taken place. The veins in the leg were varicose, but not extremely so..

Every application I could devise, every change in the treatment, appeared utterly useless. The woman would not go into an Hospital, where she might rest the limb, and perhaps have the vein obliterated by tying or caustic. I tried, therefore to find a substitute for that mode of treatment. Now the many veins on the back of the foot, and inner side of the leg converge into one trunk, the internal saphenous, which vein, with its radicles, is most frequently varicose; and I determined to apply a strong and constant pressure upon some part of that vein, to prevent the dilatation of those below, the blood finding its way through some other channel. The method used in this case was simply a firm pad, tightly fastened upon the vein, immediately beneath the head of the tibia, by two cross strips of plaster, which did not meet so as to surround the limb on the outer side. The leg was then firmly bandaged.

The effect of this treatment was almost immediate. The ulcer began to fill up, and the hard edges yielded. The pad was first used in the middle of November; by the end of February the ulcer had healed, and this while she was walking about and following her employment as a washerwoman. The other cases in which I have used this method of cure are naturally similar, and I have never found reason to dread any inconvenience whatever from the practice. A very simple instrument will, however, be found a more convenient method of compressing the vein than the cheap but rude expedient I have adopted among the poor.—*London Med. Times and Gazette.*

